# Supplementary material for: A Genome-Wide Association Study into the Aetiology of Congenital Solitary Functioning Kidney
Source: Biomedicines. 2022 Nov 23;10(12):3023. doi: 10.3390/biomedicines10123023 (PMC9775328; doi:10.3390/biomedicines10123023)
Supplement: Supplementary file 1 [file biomedicines-10-03023-s001.zip › biomedicines-1982181-supplementary.pdf]

A Genome-Wide Association Study into the Aetiology of Congenital Solitary Functioning Kidney

Sander Groen in 't Woud, Carlo Maj, Kirsten Y Renkema, Rik Westland, Tessel Galesloot, Iris ALM van Rooij, Sita H Vermeulen, Wout FJ Feitz, Nel Roeleveld, Michiel F Schreuder*, Loes FM van der Zanden*, for the SOFIA study group
*Shared last author

Quality control

AGORA cases

Of 834 unique patients, genotyping was successful in 802. After excluding cases with a known genetic cause or syndrome (n = 35) and phenotypes other than unilateral kidney agenesis, multicystic dysplastic kidney or kidney hypo/dysplasia (n = 315), 452 cases remained. In QC, two cases were removed because of sex discrepancies. In the 450 remaining cases, 686,487 variants were genotyped. During marker quality control (QC), 25,642 variants were removed due to a call rate <98%, 250 due to a failed Hardy-Weinberg test and 98,755 because of a minor allele frequency (MAF) <0.1%. Two samples were excluded due to a call rate <98%, resulting in a dataset of 448 samples and 561,840 variants.

AGORA controls and AGORA dataset

Four AGORA controls were removed because of sex discrepancies. In the resulting 665 AGORA controls, 692,998 variants were genotyped. Variants were removed due to a genotype call rate <98% (n=39,376), a failed Hardy-Weinberg test (n=1,082), or a MAF <0.1% (n=97,753), resulting in 554,787 variants passing QC. Five samples had a call rate <98% and were removed. Merging the cases with the AGORA controls resulted in a dataset with 524,412 shared variants in 1,108 individuals. Imputation yielded a database with 9,956,431 with a Minimac imputation quality score (r2) of ≥0.6 and minor allele count (MAC) of 20. After imputation, 6 cases and 12 controls were excluded due to relatedness and 39 cases and 26 controls were excluded because of non-European ancestry, leaving 403 cases and 622 controls for analysis in the AGORA dataset.

NBS controls and NBS dataset

In the NBS control group, samples with sex discrepancies and variants with a call rate <95% had been excluded previously. Of 670,542 remaining variants, 13,646 were removed due to a call rate <98%, 8,678 due to a failed Hardy-Weinberg test and 15,364 because of a MAF <0.1%, leaving 632,854 variants. Eighty-five samples were removed because of a sample call rate <98%. Only 207,692 variants were shared with the case dataset and used for imputation, which resulted in 9,313,318 SNPs with an r2 of ≥0.6 and MAC of 20. After imputation, 6 cases and 884 controls were excluded due to relatedness and 39 cases and 28 controls because of non-European ancestral backgrounds, resulting in 403 cases and 4,366 controls available for analyses in the NBS dataset.
